# Three-Dimensional Resistive Metamaterial Absorber Loaded with Metallic Resonators for the Enhancement of Lower-Frequency Absorption

**DOI:** 10.3390/ma11020210

**Published:** 2018-01-30

**Authors:** Yang Shen, Jie Qiu Zhang, Yong Qiang Pang, Lin Zheng, Jia Fu Wang, Hua Ma, Shao Bo Qu

**Affiliations:** 1 College of Science, Air Force Engineering University, Xi’an 710051, China; zhangjiq0@163.com (J.Q.Z.); 225pang@163.com (Y.Q.P.); zhenglin0205@gmail.com (L.Z.); wangjiafu1981@126.com (J.F.W.); mahuar@163.com (H.M.); Qushaobo@mail.xjtu.edu.cn (S.B.Q.); 2 School of Electronics and Information Engineering, Xi’an Jiaotong University, Xi’an 710049, China

**Keywords:** metamaterial absorber, metallic resonator, three-dimensional construction, broadband absorption, lower-frequency absorption

## Abstract

Resistive patch array incorporating with metallic backplane provided an effective way to achieve broadband metamaterial absorbers (MAs) in microwave frequency, and the outstanding construction contributed more flexible and diversified broadband absorption. In this paper, we attempted to load metallic resonators (MRs) to three-dimensional resistive MA to further enhance the lower-frequency absorption performance. Simulation showed that the partial absorption peak was separated to the lower frequency, while the rest of broadband absorption was unaffected. Meanwhile, after combining multi-unit of the proposed MAs, the stair-stepping broadband absorption was also achieved. Finally, three samples were fabricated. The agreements between simulations and experimental results demonstrated that resistive MA loaded with MRs provided an effective way for further enhancement of lower-frequency absorption with almost no change of the absorbing structure and lightweight characteristic. Thus, it was worthy to expect a wide range of applications to emerge inspired from the proposed attempt.

## 1. Introduction

Metamaterials consist of periodic subwavelength unit cell which can provide more possibility to construct the desired constitutive parameters. As new artificial medium, metamaterials have great applications in many fields, such as superlenses [1,2,3], invisibility cloaks [4,5,6], and perfect absorbers [7,8,9,10]. For the field of electrometric wave absorbing medium, the perfect metamaterial absorber was firstly proposed based on three-layered configuration of metal-dielectric-metal [7]. The strong resonance from the metallic metamaterial and the loss from the dielectric substrate worked together contributing to nearly perfect absorption at one frequency. Based on this, various MAs with one-, two- or multi-band absorption were proposed [8,9,10,11,12,13]. However, highly effective absorption is always accompanied with the narrow bandwidth, which was the fundamental obstacle limited the practical application.

To achieve broadband MAs, many attempts were carried out. First, the combination of multi-unit exciting contiguous resonances can be thought as a direct way to construct a continuous broadband absorption [14,15,16,17,18,19]. On the one hand, the multi-unit with adjacent absorption peaks can be assembled together on the same plane [14,15]. However, the inherent contradiction between the absorbing efficiency and the duty radio always hindered the further enhancement of broadband absorption. On the other hand, the varied units with the contiguous absorption peaks can also be arranged along the wave vector to achieve outstanding broadband absorption [16,17,18]. However, the expansion of absorption bandwidth was carried out at the sacrifice of ultrathin thickness and light weight characteristic. Then, loading lumped elements to MA was demonstrated to be an effective way to achieve broadband MA [19,20,21,22]. More specifically, when loading lumped resistor and capacitor to MA, several absorption peaks will not only be adjusted to more adjacent frequencies, but they also exhibit low Q factor in the spectrum. The several absorption peaks with low Q factor finally overlapped together contributing highly effective and broadband absorption. Meanwhile, with the development of fabricated technology, a series of innovated materials got a lot of attention. They were also introduced to the MA in pursuit of outstanding broadband absorption performance [23,24,25,26]. For example, resistive patch with strong ohmic loss was introduced to construct broadband MA in electromagnetic wave [27,28,29,30,31,32,33]. Compared with the aforementioned MA based on metal-dielectric-metal configuration, resistive MAs easily achieved highly effective and broadband absorption as well as other advantages, such as lightweight, low cost, and easy fabrication. However, with deep research of resistive MAs, it was also demonstrated that the further enhancement of broadband absorption, especially for lower-frequency absorption, was not easy to carry out when the total thickness of the resistive MA was given.

In this paper, aiming at the target of lower-frequency absorption enhancement in resistive MA, we attempted to load MRs to the resistive MA to reconfigure the broadband absorption performance. As a proof, the three-dimensional resistive patch array standing on the metal backplane was firstly introduced for broadband absorption. Then, the metallic bamboo joints as MRs were adhered to the resistive patches. The strong resonance inspired by the MRs at low frequency broke the original broadband absorption. Simulation showed that the proposed MA can effectively separate partial absorption peak to the lower frequency, while keeping the broadband absorption at rest frequencies unchanged. Meanwhile, after combining multi-unit, the stair-stepping broadband absorption was also achieved. Finally, three samples were fabricated for experimental demonstration. It is expected that a wide range of applications will emerge from the proposed attempt.

## 2. Three-Dimensional Resistive MA

**Figure 1:**
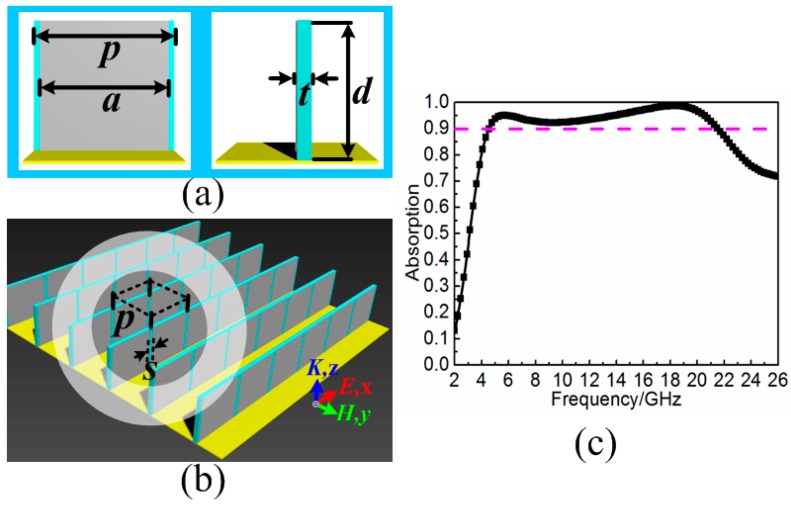
Three-dimensional resistive MA consists of resistive patches and dielectric substrates standing on the metal backplane: (**a**) front view and side view of each unit cell; (**b**) perspective view of three-dimensional resistive MA; and (**c**) absorption spectrum under normal incidence. The optimized parameters of the resistive MA unit cell are given as follows: *d* = 8.0 mm, *a* = 10.5 mm, *s* = 0.5 mm, *f_z_* = 75.0 Ω/sq, *t* = 1.0 mm, and *p* = 11.0 mm.

**Figure 2:**
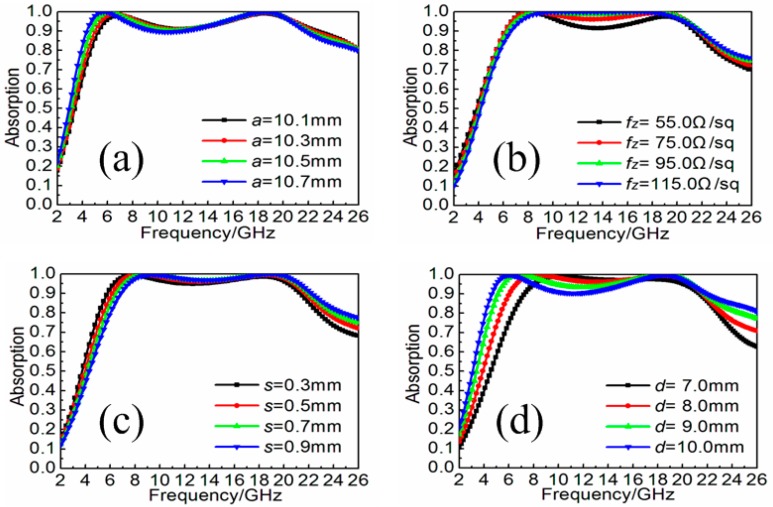
Absorption spectra of the three-dimensional resistive MAs with different: (**a**) size *a* of resistive patch ranging from 10.1 to 10.7 mm; (**b**) sheet resistance *f_z_* of resistive patch ranging from 55.0 to 115.0 Ω/sq; (**c**) gap *s* between adjacent resistive patches ranging from 0.3 to 0.9 mm; and (**d**) height *d* of resistive patch ranging from 7.0 to 10.0 mm.

Using resistive patch to construct broadband MA has been widely adopted. Here, our method attempted to use resistive patch array stand on the metal backplane as three-dimensional resistive MA. As shown in Figure 1a,b, the height, the width and the sheet resistance of each resistive patch are *d*, *a* and *fz*, respectively. The resistive patch arranged along *x*-axis direction on the square backplane with the size of *p*. The gap between adjacent resistive patches was *s*. The F4B3 substrate (WANGLIN, Taizhou, Jiangsu, China) was used here as a supporter for the construction of the resistive patch array with height *d*, width *p*, and thickness *t*. The relative permittivity and loss tangent of the F4B3 substrate are 2.65 and 0.001, respectively. The copper plate was used here as backplane with a frequency independent conductivity *σ* = 5.8 × 10^7^ S/m and the thickness *t*_c_ = 0.017 mm. In the simulation, the electric field of the incidence should be set along *x*-axis, while the magnetic field was along *y*-axis to obtain the broadband absorption. The absorptive efficiency of the three-dimensional resistive MA under normal incidence can be defined as *A*(ω) = 1 − *R*(ω) − *T*(ω) = 1 − |S_21_|^2^ − |S_11_|^2^, where *A*(ω), |S_11_|^2^ and |S_21_|^2^ are the absorbance, reflectivity and transmissivity, respectively. Due to the existence of metal backplane, the transmission (S_21_) is zero. Thus, the absorbance can be calculated by *A*(ω) = 1 − |S_11_|^2^ in this paper. After optimization, when the thickness was given as *d* = 8.0 mm, the three-dimensional resistive MA can achieve broadband electromagnetic wave absorption with efficiency more than 90% ranging from 4.6 to 21.4 GHz, as shown in Figure 1c. Meanwhile, during the optimization process, some parameters were noted to affect the broadband absorption performance, and are also discussed here. As shown in Figure 2a–d, when increasing the size *a* of resistive patch from 10.1 to 10.7 mm or decreasing the gap *s* between adjacent resistive patches from 0.9 to 0.3 mm, the broadband absorption would further move to the lower frequency with very slight extent. The sheet resistance of the resistive patch can be seen as an assistant factor for the improvement of absorption during the operating frequencies. As a comparison, the height of resistive patch played an important role in the enhancement of broadband absorption. With the increase of height from 7.0 to 10.0 mm, the low boundary frequency of absorption bandwidth with efficiency more that 90% would further expand from 7.3 to 4.6 GHz. Thus, it can be concluded that there was less space for further enhancement of lower-frequency absorption when the height of the three-dimensional resistive MA was given.

## 3. Three-Dimensional Resistive MA with MRs

**Figure 3:**
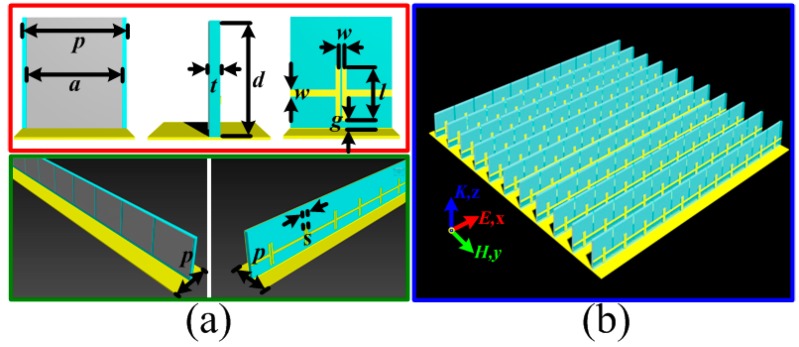
Three-dimensional resistive MA loaded with MRs: (**a**) schematic diagram of the proposed MA unit cell; and (**b**) perspective view of the proposed MA. The parameters of the proposed MA unit cell are given as follows: *d* = 8.0 mm, *a* = 10.5 mm, *s* = 0.5 mm, *f_z_* = 75.0 Ω/sq, *p* = 11.0 mm, *t* = 1.0 mm, *w* = 0.5 mm, *g* = 0.5 mm, and *s* = 0.5 mm.

**Figure 4:**
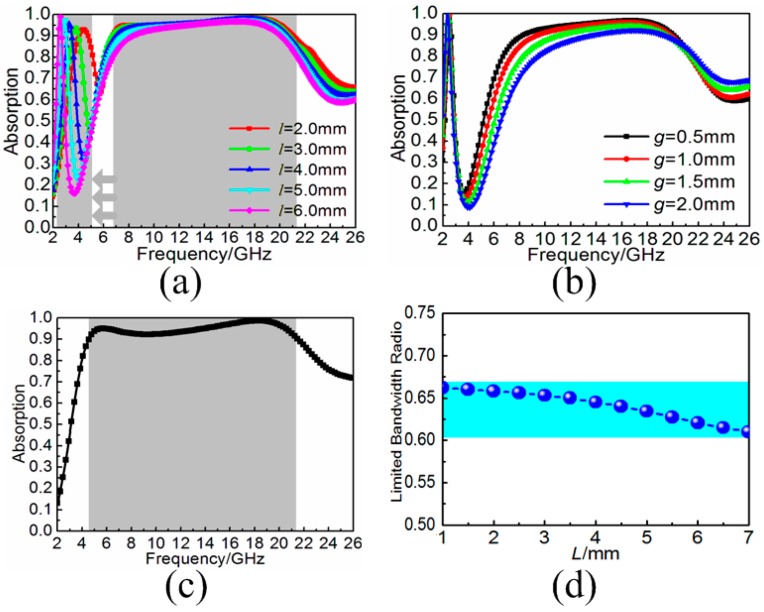
(**a**) Absorption spectra of the three-dimensional resistive MAs loaded with different widths *l* of metallic bamboo joints; (**b**) absorption spectra of the three-dimensional resistive MAs loaded with different gaps *g*; (**c**) absorption spectrum of the three-dimensional resistive MA; and (**d**) limited bandwidth radio of the three-dimensional resistive MAs with different widths *l* of metallic bamboo joints.

Here, based on the aforementioned design of three-dimensional resistive MA, we attempted to load the metallic bamboo joint as MR to reconfigure the broadband absorption. The structure diagram in shown in Figure 3a, the F4B3 substrates with height *d*, width *p* and thickness *t* were introduced here to stand up on the squared metal backplane with the size *p*. On the one side of the substrate, the aforementioned resistive patches were adhered periodically. On the other side of the substrate, metallic bamboo joint was also printed accordingly. The height, width and sheet resistance of the resistive patch are *d*, *a* and *f_z_*, respectively. The width of the wire, width of the bamboo joint, and the gap between adjacent bamboo joints are *w*, *l* and *s*, respectively. The metallic bamboo joint units were arranged along *x*-axis with the gap of *g* above the metal backplane. In the simulation, the electric field of the incidence was also set along *x*-axis to achieve optimal broadband absorption performance. When the three-dimensional resistive MA is loaded with MRs, the originally continuous broadband absorption will be affected. Instead, the partial absorption peak was separated to the lower frequency. As shown in Figure 4a, with the increased width *l* of metallic bamboo joint from 2.0 to 6.0 mm, the separated absorption peak would gradually move to the lower frequency from 4.4 to 2.4 GHz, while the continuous broadband absorption during higher frequencies was still unaffected. Here, it should be noted that optimized results demonstrated the gap *g* should be constant with metallic bamboo joint out of the center. The minimized value of *g* benefited the broadband absorption during the rest frequencies, as shown in Figure 4b. Based on the enhanced lower-frequency absorption, a generalized definition of absorption bandwidth was given which reflected the range between the lower boundary frequency of separated absorption band and the upper boundary frequency of unchanged absorption band with the efficiency more than 90%. Table 1 gave the generalized absorption bandwidth and the corresponding absorption bandwidth radio for the three-dimensional resistive MA loaded with different sized MRs. Compared with the original three-dimensional resistive MA in Figure 4c, the absorption bandwidth radio also had an obvious improvement. With the increased width *l* of the metallic bamboo joint, the absorption bandwidth radio would further increase. Thus, it was obvious that loading MRs to the three-dimensional resistive MA provided an alternative way to enhance the lower-frequency absorption, which we were more concerned with, and sacrificed the absorption in the middle frequency band, which we were less concerned with. Meanwhile, the limited bandwidth radios of the proposed MA were also calculated based on the theoretical Rozanov limit [34]. During the operating frequency band of 1.0–21.0 GHz, the aforementioned three-dimensional resistive MA achieved the limited bandwidth ratio 0.67. After loading with different sized MRs, the total absorbing efficiency had no obvious change, as given in Figure 4d. Thus, the fundamental absorbing structure was demonstrated unchanged during the broad operating frequency band. 

**Figure 5:**
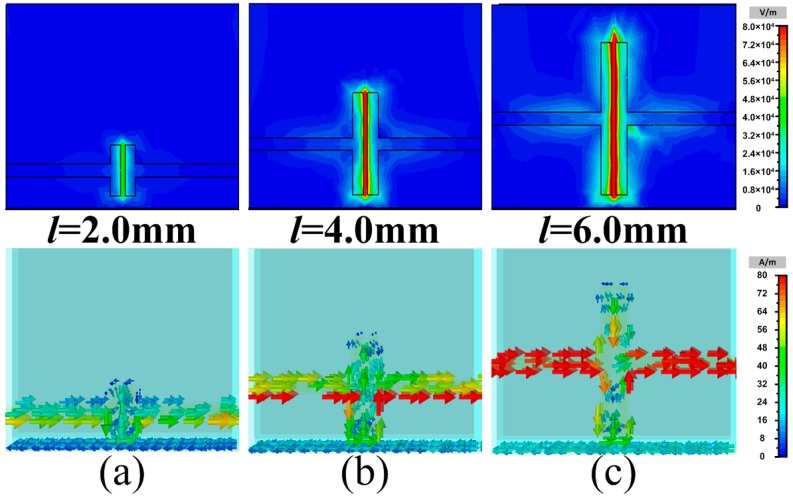
Electric field intensity distributions and surface current distributions of the proposed MAs with different widths of metallic bamboo joints: (**a**) *l* = 2.0 mm at the frequency of *f* = 4.4 GHz; (**b**) *l* = 4.0 mm at the frequency of *f* = 3.3 GHz; and (**c**) *l* = 6.0 mm at the frequency of *f* = 2.5 GHz,

**Figure 6:**
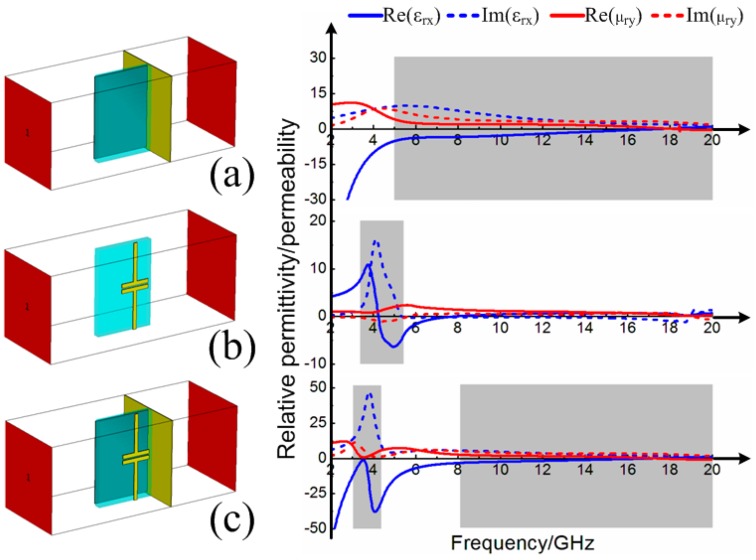
Constitutive parameters of: (**a**) the three-dimensional resistive MA; (**b**) the metallic bamboo joint on the substrate; and (**c**) the three-dimensional resistive MA loaded with the metallic bamboo joint.

**Figure 7:**
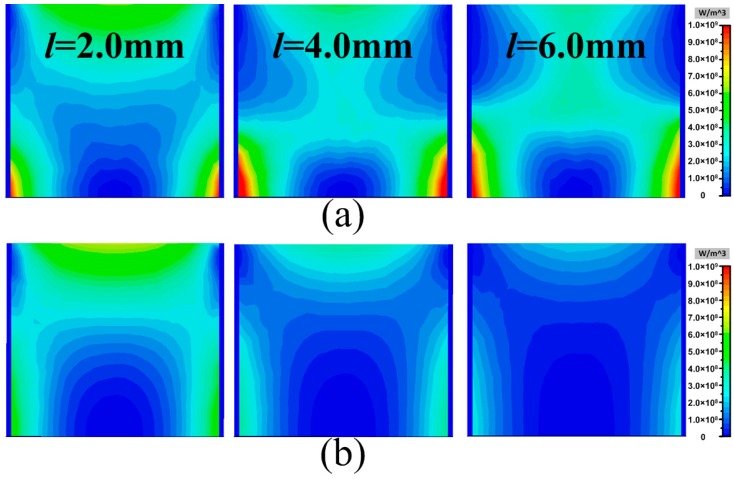
(**a**) Energy loss distributions of the proposed MAs with different widths of metallic bamboo joints (*l* = 2.0, 4.0 and 6.0 mm) at the frequencies of the lower-frequency absorption peaks (*f* = 4.4, 3.3, and 2.5 GHz, respectively); and (**b**) energy loss distributions of the three-dimensional resistive MA at corresponding frequencies.

Based on the reconfigured broadband absorption performance, the physical principles deserve exploration. Different from meta-surfaces [35] and the surface wave meta-devices [36] without consideration of loss, MA should pay more attention to the enhanced electric/magnetic field and the energy loss according to the electromagnetic power absorption in a non-magnetic medium, following the relation [37]:

$$P_{abs}=\frac{1}{2}(\omega \varepsilon^{\prime \prime }+\sigma ){\left|E\right|}^{2}$$

where *ω* is the angular frequency, *ε*″ is the imaginary part of permittivity, *σ* is the conductivity and *E* is the total electric field. First, the electric field intensity distributions and the surface current distributions of the proposed MAs with different widths *l* = 2.0, 4.0 and 6.0 mm of metallic bamboo joints at the corresponding frequency *f* = 4.4, 3.3 and 2.5 GHz of separated absorption peaks are given in Figure 5. From the electric field intensity distributions, it was obvious that the induced electric field on the three models were all enhanced at the gap of metallic bamboo joint. The corresponding surface current distributions also proved that a lot of induced surface current was generated at the gap of the metallic bamboo joint. The metallic bamboo joint was similar to electric resonator which generated strong resonance at certain frequency under normal incidence. Based on this, the metallic bamboo joint can be seen as simple RLC series circuit. The width *l* of the bamboo joint was seen as a great factor which will influence the equivalent capacitance *C* as well as the resonance frequency. Therefore, the width *l* of the bamboo joint was seen as a vital parameter directly determining the separated absorption, as shown in Figure 4a. Then, due to the loading element of metallic bamboo joint with strong resonance, the effective electromagnetic parameters of the proposed MA would bring about obvious change, which in turn affected the originally broadband impedance matching. Figure 6a firstly gives the equivalent permittivity and permeability of the three-dimensional resistive MA. Due to the low conductivity characteristic of the resistive patch itself, the resistive MA possessed flat dispersion curve, especially nearly constant in the shaded region. Figure 6b also gives the equivalent permittivity and permeability of the metallic bamboo joint printed on the F4B3 substrate. Due to the existence of the gap in the metallic unit cell, there was strong resonance in dispersion curve at low frequency in the shaded region. After combining the three-dimensional resistive MA and the metallic bamboo joint, the strong resonance at low frequency and the flat dispersion were retained, as shown in Figure 6c, which contributed the separated absorption peak at the lower frequency and the broadband absorption during high frequencies in the spectrum. FInally, the energy loss distributions of the proposed MA with different widths of *l* = 2.0, 4.0 and 6.0 mm at the frequencies of the lower-frequency absorption peaks *f* = 4.4, 3.3 and 2.5 GHz are given in Figure 7. Compared with the three-dimensional resistive MA, the energy losses were obviously enhanced at the lower frequency on the surface of resistive patches. Thus, based on the above discussion, it is worth noting that our attempt effectively enhanced the lower-frequency absorption we were particularly concerned with, while having no influence on the broadband absorption during the rest frequencies. Meanwhile, it also retains the original advantages of simple absorbing structure and lightweight characteristic.

## 4. Multi-Unit Combinations

**Figure 8:**
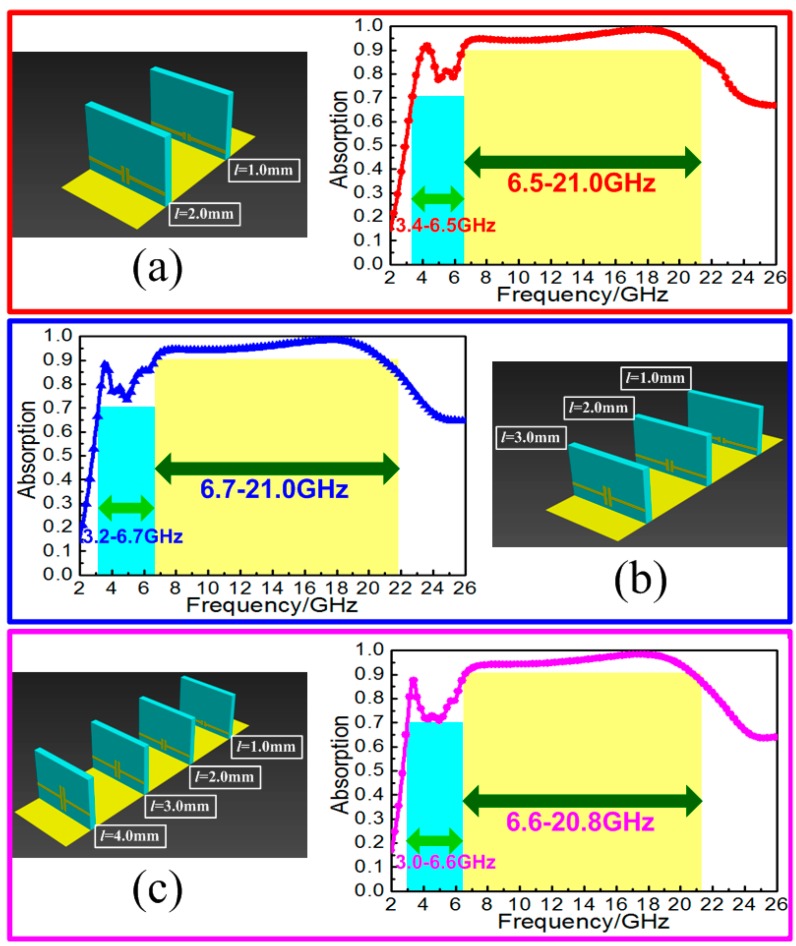
Structure diagram and simulated absorption spectra for the combination of: (**a**) two-units; (**b**) three-units; and (**c**) four-units.

Three-dimensional resistive MA loaded with MRs succeeded in separating partial absorption peak to the lower frequency for the achievement of reconfigurable broadband electromagnetic wave absorption. Due to the strong resonance inspired by the MRs, highly effective absorption band was achieved at low frequency together with the discontinuousness absorption at the middle frequencies. To eliminate the discontinuous absorption, the multi-unit combination of the proposed MA to construct the continuous broadband absorption was also proposed. Different from the similar construction applied in meta-surfaces [38] and graded-index devices [39], the proposed combined MA can be seen as a simple way to overlap the adjacent absorption peaks to construct the continuous and highly effective broadband absorption in the spectrum. Here, it should be noted that the combination of multi-unit still followed the original duty radio to keep the originally lightweight characteristic. As a proof, we constructed the combinations of two-units, three-units and four-units to discuss the broadband absorption performance. Figure 8a shows the combination of two units with the width of bamboo joints *l* = 1.0 and 2.0 mm, respectively. The simulation showed that the two resonances were excited at the frequencies of 4.2 and 5.8 GHz. A stair-stepping broadband absorption was achieved with the efficiency more than 70% from 3.4 to 6.5 GHz and 90% from 6.5 to 21.0 GHz. Figure 8b shows the combination of three units with the width of bamboo joints *l* = 1.0, 2.0 and 3.0 mm. The simulation showed that the three resonances were excited at the frequencies of 3.6, 4.4 and 6.0 GHz. A stair-stepping broadband absorption was achieved with the efficiency more than 70% from 3.2 to 6.7 GHz and 90% from 6.7 to 21.0 GHz. The combination of four-units with the widths of bamboo joints *l* = 1.0, 2.0, 3.0 and 4.0 mm is shown in Figure 8c. Although the four resonances were hard to identify in the spectrum, the absorption peaks overlapped together contributing to the continuous broadband absorption. The simulation showed that the combination of four units achieved a stair-stepping broadband absorption with the efficiency more than 70% from 3.0 to 6.6 GHz and 90% from 6.6 to 20.8 GHz. Compared with the original resistive MA, the proposed combined MA effectively expanded the broadband absorption to the lower frequency at the sacrifice of high absorbing efficiency. With the increase to multi-unit combination, continuous broadband absorption would further expand to the lower frequency, and the average absorbance would decrease accordingly. The diversified combination with desired broadband absorption would have extensive application. Meanwhile, we also calculated the limited bandwidth radio for the combination of two-units, three-units, and four-units during the frequency band of 1.0–26.0 GHz. The calculated results of the proposed three models are the same as the former resistive MA, which further verified that the fundamental absorbing structure during the operating frequencies was unchanged. Thus, three-dimensional resistive MA loaded with MRs can exhibit more flexible and various broadband absorption performances according to our expectation, which would have extensive application in the fields of stealth technology, electromagnetic compatibility, electromagnetic shield, etc.

## 5. Measurements and Discussions

**Figure 9:**
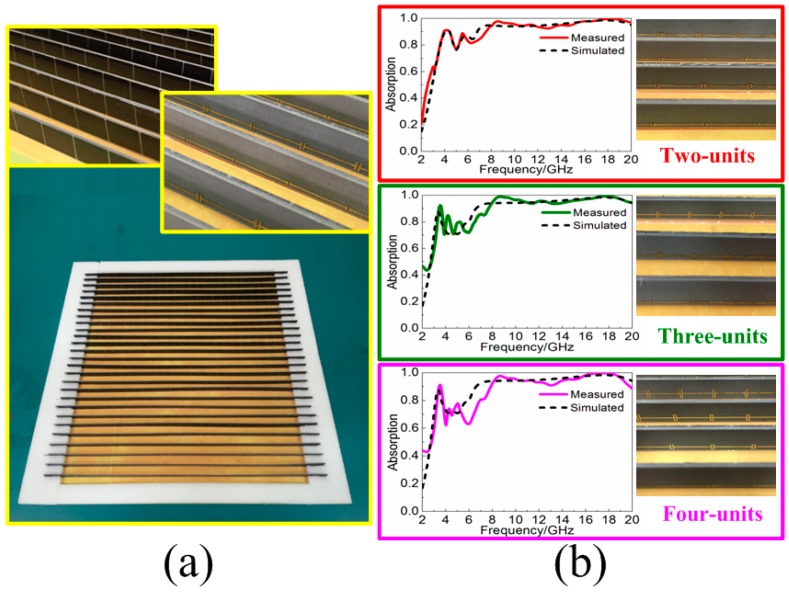
(**a**) Fabricated samples of the combined MAs in Figure 7; and (**b**) measured and simulated absorption spectra for the combination of two-units, three-units, and four-units.

To fabricate the three samples in Figure 8, the screen printing technology was firstly introduced to fabricate the resistive patch. Twenty-seven metallic bamboo joints were printed on the rectangular F4B3 substrate with the different widths *l* = 1.0, 2.0, 3.0 and 4.0 mm. Then, the resistive patches were adhered to the other side of the F4B3 substrate periodically, and the basic components of the proposed MA were achieved. Meanwhile, the implemented 297 × 297 mm^2^-sized copper plate with foam-fabricated card slots was also fabricated. The multi-unit MA components (two units, three units, and four units) were selected here and then were inserted into the card slots to construct the finished samples, as shown in Figure 9.

The experimental measurements of the fabricated three samples were performed by the arch measurement system in a microwave anechoic chamber. The system is based on an Agilent E8363B network analyzer (Santa Clara, CA, USA) with two pair of broadband antenna horns, respectively, working in the frequency bands of 2–8 GHz, 8–12 GHz, and 12–20 GHz. As shown in Figure 8b, the agreement between simulations and measurements of the three samples indicated that resistive MA loaded with MRs can effectively enhance the lower-frequency absorption with almost no change to the absorbing structure and lightweight characteristic.

## 6. Conclusions

In conclusion, we showed that resistive MA loaded with MRs can be optimized for the enhancement of lower-frequency absorption. As a proof, we loaded the metallic bamboo joint to the three-dimensional resistive MA. Simulation showed that the partial absorption peak of the proposed MA was separated to the lower frequency, while the remaining absorption band was unchanged. The separated absorption band can be adjusted with different size of MRs. Then, after combining the multi-unit of the proposed MA, the stair-stepping broadband absorption was also achieved. Finally, three samples were fabricated, and the experimental results agreed well with the simulations. Inspired by the proposed attempt, the enhancement of lower-frequency absorption is expected to be popularized in the field of electromagnetic compatibility, stealth technology, sensors, etc. Meanwhile, it should be noted that the total absorbing efficiency was not fundamentally improved during the operating frequencies. The enhancement of lower-frequency absorption was achieved at the sacrifice of the absorption during other frequencies. However, the proposed attempt provided an effective way to enhance the lower-frequency absorption of resistive MA with no effect on original absorbing structure and lightweight characteristic. Never have attempts concentrated on the reconfiguration of the broadband absorption for further enhancement of lower-frequency absorption, nor has a resistive MA loaded with MRs been designed in three-dimensional construction. In the follow-up work, the outstanding combination between the resistive MA and metallic metamaterial will be investigated for diversified and outstanding absorption performance.

## Figures and Tables

**Table 1:** Calculated absorption bandwidth radio of the three-dimensional resistive MAs loaded with different widths *l* of metallic bamboo joints.

*l* (mm)	Absorption Bandwidth (GHz)	Absorption Bandwidth Radio
2.0	4.0–21.1	1:5.3
3.0	3.5–20.7	1:5.9
4.0	3.1–20.6	1:6.6
5.0	2.7–20.3	1:7.5
6.0	2.4–20.0	1:8.3
7.0	2.3–19.8	1:8.6

Note: *l*: the width of the loaded metallic bamboo joint. Absorption bandwidth: A generalized concept that reflects the range between the lower boundary frequency of separated absorption band and the upper boundary frequency of unchanged absorption band when the absorption efficiency was more than 90%. Absorption bandwidth radio: A normalized radio between the upper boundary frequency and the lower boundary frequency.
